# Virtual Reality Exposure‐Enhanced Cognitive Behavioral Therapy (CBT + VRE) vs. CBT With Imaginal Exposure (CBT + IE) for Adolescent Patients Treatment of Social Anxiety Disorder Symptoms: A Pilot Randomized Controlled Trial

**DOI:** 10.1155/da/9956436

**Published:** 2026-05-25

**Authors:** Parisa Azimisefat, Samin Ravanbod, Ad de Jongh, Maryam AdliHamzehkhanlou, Fateme Jamshidi, Yousef Dehghani, Farzaneh Yazdani, Philipp Herzog

**Affiliations:** ^1^ Department of Psychology, Persian Gulf University, Bushehr, Iran, pgu.ac.ir; ^2^ Medical Biology Research Center, Health Technology Institute, Kermanshah University of Medical Science, Kermanshah, Iran, kums.ac.ir; ^3^ Research Department, PSYTREC, University of Amsterdam and VU University Amsterdam, Bilthoven, Netherlands, vu.nl; ^4^ School of Psychology, Queen’s University, Institute of Health and Society, University of Worcester, Belfast, Northern Ireland, UK, worcester.ac.uk; ^5^ Department of Educational Science, Ataturk University, Erzurum, Türkiye, atauni.edu.tr; ^6^ Department of Psychology, University of Kaiserslautern–Landau (RPTU), Landau, Germany

**Keywords:** adolescents, cognitive behavior therapy, fear of negative evaluation, social anxiety disorder, virtual reality exposure

## Abstract

**Background:**

The purpose of the present study was to investigate the relative effectiveness of cognitive–behavioral therapy (CBT) with imaginal exposure (CBT + IE) and CBT combined with virtual reality exposure (CBT + VRE) for symptoms of social anxiety disorder (SAD) and fear of negative evaluation (FNE) in adolescents.

**Methods:**

In a three‐armed randomized controlled trial (RCT), a total of 51 adolescents who met the DSM‐5 criteria for SAD were randomly assigned to either CBT + IE (*N* = 17; *M*_age_ = 16.41; *SD* = 0.61), CBT + VRE (*N* = 17; *M*_age_ = 16.35; *SD* = 0.70), or a waitlist control condition (WLCC; *N* = 17; *M*_age_ = 16.00; *SD* = 0.70). Participants were assessed pre and posttreatment and followed up 3 months after the last treatment session regarding symptoms of social phobia, measured with the social phobia inventory (SPIN), and FNE, measured with the FNE Questionnaire.

**Results:**

A linear mixed model (LMM) analysis revealed significant differences between the treatment groups and WLCC in interaction with the three assessment times in SAD and FNE. A significant reduction of both CBT + VRE and CBT + IE treatments on symptoms of SAD from pretreatment to posttreatment and 3 months follow‐up (*d*_pre–post_ = 1.31 and *d*_pre-fu_ = 1.43 in CBT + VRE group; *d*_pre–post_ = 1.90 and *d*_pre-fu_ = 2.04 in CBT + IE group) and FNE (*d*_pre–post_ = 1.35 and *d*_pre-fu_ = 1.68 in the CBT + VRE group; *d*_pre–post_ = 1.95 and *d*_pre-fu_ = 1.59 in the CBT + IE group) compared to the WLCC was observed. No significant differences were observed between the VRE + CBT and CBT + IE groups on FNE. With respect to SAD, statistically significant between‐group differences emerged in favour of the CBT + IE group, accompanied by substantial effect sizes at posttreatment (*d* = 0.87) and follow‐up (*d* = 0.73).

**Conclusion:**

The results support the effectiveness of CBT + IE and CBT + VRE in reducing the symptoms of SAD and FNE, which were maintained 3 months after treatment. Future research should focus on optimizing VRE protocols, exploring long‐term outcomes, and investigating its applicability across diverse populations. Further exploration of the cost‐effectiveness and accessibility of VR technology in clinical settings is required.

**Trial Registration:** ClinicalTrials.gov identifier: IRCT20210213050343N2

## 1. Introduction

Social anxiety disorder (SAD), as defined in the DSM‐5‐TR, is characterized by a marked fear or anxiety about one or more social situations in which individuals are exposed to possible scrutiny by others 1]. SAD is an early‐onset and often chronic mental health condition [2, 3]. Epidemiological studies indicate that while the prevalence of a SAD diagnosis among adolescents typically ranges from 2.0% to 5.7% [4], up to 36% of young people may report significant social anxiety symptoms that do not necessarily meet the full diagnostic criteria [5, 6], with one study specifically focusing on adolescents aged 12–17 years [6]. SAD has been found to increase the risk of co‐occurring psychiatric disorders such as substance use disorder, major depressive disorder, and other mood and anxiety disorders [7]. In addition, SAD is associated with an increased risk of cardiovascular disease and social dysfunction [7].

Cognitive–behavioral models identify fear of negative evaluation (FNE) as the core feature of SAD [8]. Yet, research suggests that individuals with SAD may fear both negative and positive evaluation [9]. The bivalent fear of evaluation model posits that social threat arises whenever one is observed and judged by others. Anticipated negative evaluation fosters concerns about rejection and exclusion, whereas anticipated positive evaluation may also be distressing, as it heightens expectations and perceived performance demands, often culminating in fears of failure [10]. Sensitivity to both forms of evaluation contributes to the persistence of social anxiety, exacerbating distress and avoidance [11]. These patterns frequently impair academic, occupational, and interpersonal functioning [10]. Given the pervasive impact of SAD, the identification and dissemination of effective treatments remain a clinical priority (American Psychiatric [1].

The first line of treatment for anxiety disorders is cognitive–behavioral therapy (CBT), a diverse group of interventions that targets the three main dimensions of anxiety disorders [12]. These dimensions include cognitive (e.g., cognitive distortions about the possibility of being harmed), behavioral (e.g., avoiding potentially stressful situations), and physiological dimensions (e.g., stimulation of the autonomic nervous system and other physical symptoms [13]. The main cornerstone of CBT for SAD is exposure to anxiety‐inducing social situations in order to challenge their anxiety‐related expectations ([14–17].

A relatively new form of exposure therapy that has been studied for SAD is virtual reality exposure therapy (VRET) [16, 17]. In VRET, participants are exposed to computerized stimuli such as virtual social interactions, which can induce high levels of social anxiety. Research has shown that virtual reality therapy is effective in treating a wide variety of anxiety disorders [18]. Randomized controlled trials (RCTs) investigating the effectiveness of VRE therapy, both alone and in combination with CBT, for SAD reported positive results for these treatments [19–21]. Despite growing interest in VRET, RCTs (RCTs) examining its efficacy in SAD remain scarce, and most have focused on adults [22, 23]. These trials typically compared VRET with in vivo exposure, often in combination with cognitive interventions, and generally reported comparable outcomes across conditions [24, 25]. Although such findings support the effectiveness of VRET in adults, little is known about its utility for children and adolescents, despite the early onset and chronic trajectory of SAD [26]. A further limitation is that few studies have contrasted VRET with imaginal exposure (IE), a modality frequently used in clinical practice. While in vivo exposure is the gold standard, it faces practical barriers: recreating feared scenarios can be time‐consuming, resource‐intensive, and difficult to replicate consistently. IE circumvents these challenges but relies on patients’ capacity and willingness to vividly imagine feared situations, a demand that may be particularly problematic for younger populations. In contrast, VRET offers immersive, standardized environments that reliably elicit strong emotional responses and may foster greater engagement and therapeutic gains [27, 28]. The interactive and technologically appealing nature of VR may further enhance adolescents’ motivation to participate in exposure exercises [25, 29].

To address these issues and gaps, the purpose of this pilot study was to determine the feasibility and effectiveness of structured, clinic‐based VRET (CBT + VRE) compared to IE (CBT + IE) in adolescents with SAD on SAD symptoms and FNE among adolescents. We hypothesized that patients treated with CBT + VRE would show a significantly greater reduction in symptoms of SAD and FNE, compared to patients treated with CBT + IE, and that this reduction would persist at the 3‐month follow‐up.

## 2. Materials and Methods

### 2.1. Study Design

**Figure 1:**
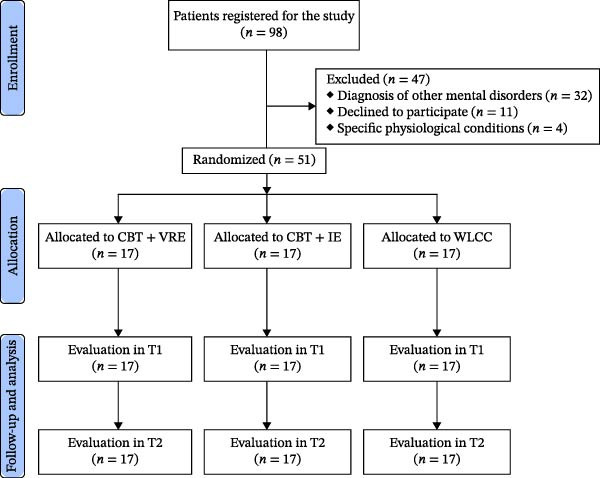
Participant flowchart.

We conducted a pilot randomized controlled study involving 51 adolescent patients at the Department of Psychology of Persian Gulf University (PGU). The study had a parallel design and was approved by the Research Ethics Committees of Bushehr Province University of Medical Science (Reference: IR.BPUMS.REC.1402.124). Participants were assigned to one of three groups: (I) CBT + VRE, (II) CBT + IE, and (III) waiting list control condition (WLCC). Each group consisted of 17 adolescent patients who were aware of their group assignments owing to the nature of the interventions. The assessors remained blind to group assignments. The interventions carried out by Parisa Azimisefat (the first author) followed the TIDierR checklist [16]. The study design is shown in Figure 1 (CONSORT flowchart).

### 2.2. Participants

Participants were recruited through referrals from school counselors and local children’s and adolescents’ mental health clinics in Bushehr, as well as through advertisements posted on the PGU website and social media platforms. Families who expressed interest were contacted by phone and invited to the Department of Psychology at PGU for an initial screening interview. Participants were eligible for inclusion in the study if they met all the diagnostic criteria for SAD based on the Structured Clinical Interview for DSM‐5 (SCID‐5) [30]. They had to be aged between 14 and 18 years, and provided written informed consent completed by individuals and their parents. The criteria for excluding participants from our study were the presence of vertigo, hearing or visual impairments (such as stereoscopic blindness or nystagmus), addiction to alcohol or drugs, specific physiological conditions (such as heart, lung, and respiratory diseases, epilepsy, and seizures), developmental or intellectual disability, cognitive impairment, fulfillment of the diagnostic criteria of any other mental health condition as indexed by the SCID‐5, or receipt of other current psychological treatments.

An independent clinician administered the SCID‐5 [30] to confirm the diagnosis of SAD and to screen for comorbid psychiatric conditions. Exclusion criteria such as substance use, neurological disorders, and medical conditions (e.g., epilepsy and cardiovascular disease) were assessed via self‐report and medical history forms. Intellectual disability and cognitive impairment were screened through clinical interview with both the adolescent and parents, corroborated by available school and medical records.

### 2.3. Procedure

All participants and their parents received detailed information about the study and completed and signed a consent form. The participants were assigned to different groups based on a randomized, balanced block method using a random number table CBT + VRE (*n* = 17), CBT + IE (*n* = 17), and WLCC (*n* = 17). The randomization method was simple and the randomization unit was individual. The randomization sequence was generated by the consultant epidemiologist in Microsoft Excel (Microsoft Corporation, Redmond, WA, USA) using the RAND function to produce four balanced blocks. This ensured that participants were evenly allocated to the three groups (CBT + VRE, CBT + IE, and WLCC). The allocation list was prepared in advance by the epidemiologist. Neither the therapist nor participants were blind. However, data analysts and outcome assessors were blinded to the participants’ group assignments. Before the first session, the researcher collected information regarding the patient’s medical history. The researcher explained the purpose of the study to the patients and planned the future treatment sessions. Participants received twelve 60 min treatment sessions twice per week in both experimental conditions (CBT + VRE and CBT + IE). Assessments were carried out 1 week before (pretreatment), 1 week after (posttreatment), and 3 months after the last treatment session (follow‐up). Only data from participants who completed the questionnaires on social anxiety and FNE at all three assessment points were included in the analyses.

### 2.4. Interventions

#### 2.4.1. Virtual Environments for Social Anxiety Disorder

**Figure 2:**
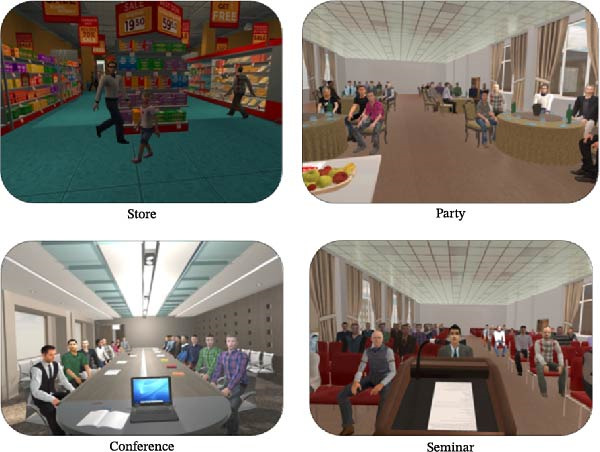
VR scenarios.

In the present study, the CBT + VRE intervention was performed using a desktop computer equipped with specific components ASUS NVIDIA GEFORCE GTX 1060 6 GB GDDR5 Graphics Card, Core i7−4790 CPU (8M Cache, up to 4.00 GHz), 8 GB DDR4 RAM, and an Oculus Rift Dk2 Virtual Reality Headset. These devices allowed us to create a virtual environment that delivered VR stimuli safely to the patient. Figure 2 shows the VR scenarios used in this study. Four different VR scenarios are available as follows:

• Store: the patient is shopping in a virtual store. Sellers and other people are also present in the store where the patient can approach.
• Party: the patient participates in a big party and has to perform a speech or poem for the audience.
• Conference: the patient is taking part in an official meeting in which she/he must give a speech to the audience about her/his field of study and maintain eye contact with them.
• Seminar: the patient is attending a seminar with a large number of participants. She/he must present the text that she/he prepared beforehand at the lectern in front of the audience. She/he is asked to address the audience in that seminar, keeping eye contact with them.

Table 1 summarizes the key components and delivery modes of treatment protocols for SAD. We compared the CBT + VRE protocol, which incorporates virtual reality exposure, with a standard CBT protocol. The primary difference lies in the delivery mode, with CBT + VRE utilizing virtual exposure and CBT protocol relying solely on IE. In the CBT + VRE and CBT + IE groups, the intervention was conducted with a protocol based on the Clark and Wells model [8]. In the final CBT + VRE session, all VRE scenarios were presented consecutively. The VRE scenarios used in the CBT + VRE protocol were developed by the Cognitive–Behavioral Sciences Research Institute using the Unity platform [27]. Participants were able to actively navigate the virtual environments using standard VR controllers, with smooth locomotion allowing them to approach, avoid, or explore the social situations presented. The virtual characters in the scenarios exhibited preprogrammed social responses (e.g., reacting to the participant’s proximity or gaze). Therapists had control over the exposure settings both before and during the session, including selecting scenarios, adjusting social intensity (e.g., audience size and verbal engagement), and pausing or repeating segments to optimize exposure exercise.

**Table 1:** Treatment protocols for CBT + IE and CBT + VRE.

Session (duration: 60 min. each arm)	CBT + VRE	CBT + IE
1	Introduction to therapy: providing an overview of the therapy process Goal setting and problem identification: collaboratively defining therapy objectives and identifying specific issues Therapy contract signing: formalizing the therapeutic agreement Homework assignments: assigning tasks or exercises to reinforce progress outside of therapy sessions	

2	Educating about social anxiety by providing insights and knowledge about it Assigning homework tasks	

3	Explaining and introducing the cognitive model of anxiety to the patient Assigning homework tasks	

4	Understanding cognitive distortions: educating about distorted thought patterns Introducing cognitive restructuring: incorporating strategies to challenge negative automatic thoughts Assigning homework tasks	

5	Behavioral techniques introduction: familiarizing patients with practical behavioral strategies Assigning homework tasks	

6–11	Virtual exposure Exposing fears through exposure: ‐ Talking about gradually confronting anxiety‐triggering situations, consisting of psychoeducation about why we do exposure, and encouragement to do exposure during the session. ‐ Discussing safety behaviors that may inadvertently maintain anxiety and conducting behavioral experiments to test the validity of participants’ negative predictions (e.g., deliberately dropping or modifying safety behaviors such as avoiding eye contact, speaking softly, or overpreparing). The emphasis of exposure tasks was on expectancy violation and testing maladaptive predictions, rather than solely on fear reduction. Unraveling self‐beliefs by exploring how one perceives oneself in social contexts and engaging in cognitive reformulation to challenge and reshape those beliefs. Assigning homework tasks to consolidate in‐session learning, including continued exposure practice and behavioral experiments in real‐life contexts.	Imaginal exposure Patients were encouraged to repeatedly verbalize their primary worry thoughts, like “I will make a mistake, and others will judge me.” In addition, they were tasked with writing a concise narrative (3–5 sentences) where their fears came true, which they then read aloud multiple times. Sometimes, we adapted the process based on individual needs, having patients either speak their thoughts, write narratives, or combine both methods. Exposing fears through exposure: ‐ Talking about gradually confronting anxiety‐triggering situations; consisting psychoeducation about why we do exposure, and encouragement of doing exposure during the session. ‐ Discussing safety behaviors that may inadvertently maintain anxiety Unraveling self‐beliefs: ‐ Exploring how one perceives themselves in social contexts ‐ Engaging in cognitive reformulation to challenge and reshape those beliefs Assigning homework tasks

12	Therapy summary	

After being randomly assigned, WLCC patients were scheduled for a 12‐week follow‐up assessment. If their symptoms deteriorated during the waiting time, patients could reach the researcher via telephone. Following the intervention phase of the study, WLCC patients had the opportunity to receive treatment from psychologists at the university clinic.

### 2.5. Assessment and Outcome Measures

#### 2.5.1. Social Phobia Inventory (SPIN)

The SPIN is a self‐report scale designed to screen and measure SAD. This instrument comprises 17 items that assess fear, avoidance, and physiological discomfort in social or performance situations over the previous week. SPIN is rated on a five‐point Likert scale ranging from 0 (not at all) to 4 (extremely). Higher total SPIN scores indicate greater severity of SAD [31].

#### 2.5.2. Brief Version of the FNE (BFNE) Questionnaire

The BFNE is a brief version of the FNE scale. This scale helps assess social anxiety and fear of judgment and provides insights into how individuals perceive themselves in social situations. The items focus on apprehension related to being evaluated negatively by others [32]. Participants rate each of the 12 items on a five‐point Likert scale to express the degree of their FNE.

### 2.6. Data Analysis

The primary aim of this pilot study was to evaluate variability and generate preliminary effect‐size estimates to guide the design of future large‐scale trials. Variability was examined through standard deviations and confidence intervals of primary outcome measures across groups and time points, which can help determine sample size estimations for future trials and were crucial for refining trial procedures. To compare the two groups, a linear mixed model (LMM) was utilized with post hoc comparisons using the least significant difference (LSD) test. Additionally, to validate the findings and offer more details about clinically meaningful differences, ANOVA was conducted. In addition to statistical significance testing, effect sizes are reported as Cohen’s *d* throughout the manuscript to quantify the magnitude of within‐ and between‐group differences in a standardized and clinically interpretable manner. A total of 51 participants (17 per group) were recruited, based on both practical considerations and effect sizes reported in similar studies. In addition, an exploratory post hoc power analysis was performed using the observed effect sizes in this study (Cohen’s *d* = 1.90 for the CBT + IE group and Cohen’s *d* = 1.31 for the CBT + VRE group from pre‐ to posttreatment). Simulation analyses suggested that the study had adequate sensitivity to detect large treatment effects. Between‐group comparisons were conducted using the per‐protocol principle, excluding participants with missing data. Within‐group comparisons between baseline and follow‐up values and between‐group differences at follow‐up were corrected for multiple tests using the Holm–Bonferroni method. In the LMM analysis, the Sidak method was used to correct for multiple comparisons. The covariance structure that provided the best model fit was chosen based on Akaike’s Information Criterion (autoregressive). Model selection was conducted for each outcome. The main parameter of interest was the group‐by‐time interaction, indicating a different outcome pattern overtime among the three groups. Although the LSD test was initially used for pairwise comparisons, the results were interpreted with Holm–Bonferroni corrections applied to control for inflated type I errors. Data analysis was performed using SPSS version 27.

## 3. Results

### 3.1. Demographics

Table 2 presents the participants’ sociodemographic characteristics. Participants were on average 16 years old (*M*_age_ = 16.35, SD = 0.70 for CBT + VRE; *M*_age_ = 16.41, SD = 0.61 for CBT + IE; and *M*_age_ = 16.00, SD = 0.70 for WLCC). One‐way ANOVA indicated no significant differences in age between the CBT + VRE, CBT + IE, and WLCC groups (*F* = 1.840, *p* = 0.10). Chi‐square tests revealed no significant differences in the other demographic variables among the groups. Baseline symptom severity was assessed using the SPIN for SAD and the BFNE for FNE (Table 2). A one‐way ANOVA revealed no significant differences in baseline symptom severity across conditions for either SPIN scores, *F* (2, 48) = 0.43, *p* = 0.65, or BFNE scores, *F* (2, 48) = 1.59, *p* = 0.21. These results indicate that the groups were comparable in terms of initial symptom severity before intervention. Chi‐square tests revealed no significant differences in the other demographic variables among the groups.

**Table 2:** Sociodemographic characteristics of the participants.

Descriptive characteristics	CBT + VRE	CBT + IE	WLCC	ANOVA	Chi‐square	*p*
Age M (SD)	16.35 (0.70)	16.41 (0.61)	16.0 (0.70)	1.84	—	0.170
Gender N (%)	—	—	—	—	0.158	0.924
Female	7 (41.2)	8 (47.1)	8 (47.1)	—	—	—
Male	10 (58.8)	9 (52.9)	9 (52.9)	—	—	—
Baseline SAD M (SD)	45.05 (2.88)	44.35 (3.01)	45.23 (2.86)	0.43	—	0.65
Baseline FNE M (SD)	36.64 (6.19)	35.94 (5.99)	39.23 (4.69)	1.59	—	0.21
Mother working outside home N (%)	—	—	—	—	0.160	0.923
Yes	7 (41.17)	7 (41.17)	8 (47.5)	—	—	—
No	10 (58.82)	10 (47.1)	9 (52.94)	—	—	—
History of referring to psychologist N (%)	—	—	—	—	2.125	0.346
Yes	15 (88.2)	16 (94.1)	17 (100)	—	—	—
No	2 (11.8)	1 (5.9)	0 (0)	—	—	—

Abbreviations: CBT + IE, cognitive–behavioral therapy with imaginal exposure; FNE, fear of negative evaluation; M, mean; N, number; SAD, social anxiety disorder; SD, standard deviation; VRE, virtual reality exposure; WLCC, waiting list control condition.

### 3.2. Analysis of Outcome Measures

**Figure 3:**
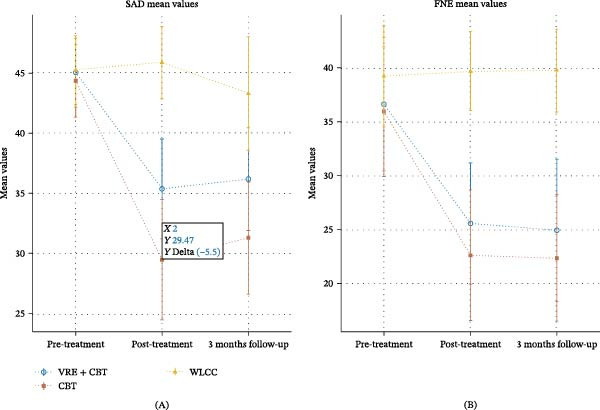
(A) Mean of SAD symptoms scores at the different measurement times from pretreatment to follow‐up for each condition, (B) fear of negative evaluation (FNE) mean scores at pretreatment to follow‐up for each condition. Error bars represent standard errors of the means.

A LMM analysis revealed a significant overall reduction in SAD scores following treatment, with a significant interaction across three assessment time points for both treatment conditions (VRE + CBT: *F* [1, 32] = 72.205, *p*  < 0.001; CBT + IE: *F* [1, 32] = 135.170, *p*  < 0.001) compared to the waitlist control condition (WLCC), and there were significant differences between the VRE + CBT and CBT + IE groups (*F* [1, 32] = 13.920, *p*  < 0.001). Also, there was a significant overall reduction in FNE scores following treatment, with a significant interaction across three assessment time points for both treatment conditions (VRE + CBT: *F* [1, 32] = 64.857, *p*  < 0.001; CBT + IE: *F* [1, 32] = 87.423, *p*  < 0.001) compared to the waitlist control condition (WLCC), and no significant differences between the VRE + CBT and CBT + IE groups (*F* [1, 32] = 0.24, *p* = 0.058) were found (Tables 3 and 4). The changes in the mean SAD and FNE symptoms at different measurement times are presented in Figure 3. Additionally, a post hoc power analysis was performed for the between‐group comparison (CBT + VRE vs. CBT + IE) on the primary outcome (SAD symptoms, SPIN). Based on the observed effect size (Cohen’s *d* = 0.30), the achieved power was 0.22 (*α* = 0.05, two‐tailed). This indicates that the study was underpowered to detect small‐to‐moderate between‐group effects, consistent with its feasibility nature (Table 5). The results of the ANOVA for between‐group comparisons indicated significant differences between the CBT + VRE and WLCC groups in posttest and follow‐up scores for both SAD (posttest: *F* = 72.20, *η* ^2^ = 0.69; follow‐up: *F* = 21.33, *η* ^2^ = 0.40) and FNE (posttest: *F* = 64.85, *η* ^2^ = 0.67; follow‐up: *F* = 63.99, *η* ^2^ = 0.66). Similarly, significant between‐group differences were observed between the CBT + IE and WLCC groups at both posttest and follow‐up for SAD (posttest: *F* = 135.17, *η* ^2^ = 0.80; follow‐up: *F* = 55.71, *η* ^2^ = 0.63) and FNE (posttest: *F* = 98.76, *η* ^2^ = 0.75; follow‐up: *F* = 87.42, *η* ^2^ = 0.73). In the comparison between CBT + VRE and CBT + IE, no significant differences were found for FNE at either posttest or follow‐up (posttest: *F* = 3.86, *η* ^2^ = 0.10; follow‐up: *F* = 0.24, *η* ^2^ = 0.00). However, for SAD, significant differences between these two groups were observed at both posttest (*F* = 13.92, *η* ^2^ = 0.30) and follow‐up (*F* = 10.03, *η* ^2^ = 0.23).

**Table 3:** Parameter estimates of the fixed effects of the LMM analysis.

Measures	SAD					Fear of negative evaluation				
Parameter	Value	SE	*p*‐Value	CI		Value	SE	*p*‐Value	CI	
				Lower	Upper				Lower	Upper
Intercept^a^	45.235	0.750	<0.001	43.702	46.769	35.235	1.605	<0.001	36.012	42.458
Time (vs. pretest)										
Posttest	7.980	0.675	<0.001	6.633	9.328	7.627	0.863	<0.001	5.902	9.353
3‐month follow‐up	7.922	0.716	<0.001	6.490	9.353	7.745	0.871	<0.001	6.002	9.488
Group										
CBT + VRE vs. CBT + IE	3.863	0.833	<0.001	2.192	5.533	1.902	1.593	0.239	−1.304	5.108
CBT + VREvs. WLCC	−5.902	0.833	<0.001	−7.572	−4.232	−10.176	1.593	<0.001	−13.382	−6.971
CBT + IE vs. WLCC	−9.765	0.833	<0.001	−11.435	−8.094	−12.078	1.593	<0.001	−15.284	−8.873
Time × Group.										
Posttest × group	10.941	1.386	<0.001	3.096	8.669	22.74	1.763	<0.001	0.408	9.585
Follow‐up × group	8.000	1.503	<0.001	1.974	8.026	10.588	1.786	<0.001	2.509	11.255

Abbreviations: CBT + IE, cognitive–behavioral therapy with imaginal exposure; SAD, social anxiety disorder; VRE, virtual reality exposure; WLCC, waiting list control condition.

^a^Reference group (intercept): waiting list control condition at pretreatment.

**Table 4:** Change in symptoms overtime by treatment condition.

Measures	SAD						Fear of negative evaluation					
Parameter	*t* [16]		*p*‐Value		*d*		t [16]		*p*‐Value		*d*	
	Pre to post	Pre to follow‐up	Pre to post	Pre to follow‐up	Pre to post	Pre to follow‐up	Pre to post	Pre to follow‐up	Pre to post	Pre to follow‐up	Pre to post	Pre to follow‐up
CBT + VRE	6.661	5.916	<0.001	<0.001	1.616	1.435	7.216	6.936	<0.001	<0.001	1.750	1.682
CBT + IE	9.123	8.429	<0.001	<0.001	2.213	2.044	6.907	6.589	<0.001	<0.001	1.675	1.598
WLCC	−0.839	1.997	0.414	0.063	−0.203	0.484	−0.739	−1.144	0.470	0.269	−0.179	−0.277

Abbreviations: CBT + IE, cognitive–behavioral therapy with imaginal exposure; SAD, social anxiety disorder; VRE, virtual reality exposure; WLCC, waiting list control condition.

**Table 5:** Between‐group comparison of symptom changes of the ANOVA analysis.

Measures	SAD						Fear of negative evaluation					
Parameter	*F* (1,32)		*p*‐Value		*d*		*F* (1,32)		*p*‐Value		*d*	
	Posttreatment	Follow‐up	Posttreatment	Follow‐up	Posttreatment	Follow‐up	Posttreatment	Follow‐up	Posttreatment	Follow‐up	Posttreatment	Follow‐up
CBT + VREvs. WLCC	72.205	21.331	<0.001	<0.001	2.01	1.12	64.857	63.992	<0.001	<0.001	1.78	1.69
CBT + IE vs. WLCC	135.170	55.711	<0.001	<0.001	3.80	2.04	98.767	87.423	<0.001	<0.001	2.67	2.52
CBT + VRE vs. CBT + IE	13.920	10.037	<0.001	0.003	0.87	0.73	3.869	0.245	0.058	0.624	0.47	0.14

Abbreviations: CBT + IE, cognitive–behavioral therapy with imaginal exposure; SAD, social anxiety disorder; VRE, virtual reality exposure; WLCC, waiting list control condition.

## 4. Discussion

The present study aimed to investigate the efficacy of CBT + VRE and CBT + IE on the symptoms of SAD and FNE among adolescent patients. Baseline assessments confirmed no significant differences among the three groups (CBT + VRE, CBT + IE, WLCC) in terms of SAD and FNE severity, ensuring comparability at the outset. The results indicated that both CBT + VRE and CBT + IE were associated with significantly reduced symptoms of SAD and FNE in comparison to the waiting list condition among patients. Furthermore, CBT + IE resulted in a substantially greater reduction in social anxiety symptoms compared to CBT + VRE. Conversely, contrary to our hypothesis, no significant difference was found between the two treatments in reducing FNE.

The observed reductions in SAD symptoms and FNE are consistent with the established efficacy of CBT for SAD and reinforce exposure as a central mechanism of change [8, 14]. The large within‐group effect sizes for both CBT + VRE and CBT + IE align with prior trials in adult populations, demonstrating that VRE is at least comparable to traditional exposure modalities [23, 25]. More specifically, consistent with previous studies [25, 29], patients who received CBT + VRE demonstrated significant reductions in SAD and FNE symptoms compared to the waiting list condition. These findings corroborate evidence that therapist‐guided virtual environments enable patients to confront socially challenging situations in a safe and controlled manner, providing opportunities for expectancy violation and corrective learning [33]. Extending this literature, the present study provides much‐needed evidence that VRE is also effective and feasible in adolescent populations, a group for whom empirical data remain scarce despite the early onset and chronicity of SAD.

Although both treatment conditions yielded significant symptom reduction, CBT + IE proved superior to CBT + VRE, a finding that contrasts with prior studies reporting comparable efficacy [25, 34]. While our study was not designed to directly test mechanisms of change (e.g., enhancing tolerance of uncertainty, fostering emotional regulation, and reducing reliance on others’ evaluations), the greater efficacy of CBT + IE compared to CBT + VRE may be explained by differences in how exposure tasks addressed patients’ fears. Conceivably, IE may have enabled highly personalized scenarios that directly captured idiosyncratic social concerns, thereby maximizing expectancy violation and facilitating cognitive restructuring. In contrast, the standardized VR scenarios, although immersive, may not have fully represented participants’ most salient fears, potentially limiting their ecological validity.

The differential efficacy between CBT + IE and CBT + VRE in reducing SAD symptoms may be related to developmental characteristics and methodological differences between the exposure modalities. Our adolescent sample differed from that of previous clinical trials involving adults. Conceivably, certain developmental characteristics, such as heightened internal emotional awareness and a rich capacity for mental imagery, may have allowed adolescents to engage particularly deeply with the IE exercises. In contrast, the more externally focused, multisensory nature of VRE, while still engaging, might have offered a different (and in this case, slightly less potent) pathway for cognitive and emotional change within this specific adolescent sample. In addition to the developmental stage, treatment effects may depend on clinical context and the flexibility of exposure tasks. While VRE offers standardized and immersive environments, its scenarios often lack the unpredictability and nuance of real‐life interactions, and technical limitations restrict to some extent the realism of social responses [25, 35]. This interpretation is supported by Wechsler et al. [36], who found that in vivo exposure was superior to VR exposure specifically in SAD, but not in agoraphobia or specific phobias. They suggest that the difficulty of simulating naturalistic social interactions in VR, combined with the complexity and chronicity of SAD, may limit the efficacy of VR‐based interventions [36]. In contrast, IE allows therapists and patients to collaboratively construct highly individualized scenarios, adapt intensity in real time, and directly target personally relevant fears. This flexibility may explain why CBT + IE produced greater improvements in both SAD and FNE in our study. Hence, although this study compared VRE and IE rather than including in vivo exposure, this design choice reflects the study’s aim to address an underexplored comparison in the literature. As in vivo exposure remains the predominant exposure modality in most CBT manuals for SAD, our findings are best generalized to treatment protocols emphasizing imaginal or virtual exposure. This focus may provide novel insight into how these two controlled and replicable modalities may differentially impact adolescent patients suffering from SAD.

This study has several limitations. First, outcomes relied exclusively on self‐report measures without clinician‐rated assessments, such as the Liebowitz Social Anxiety Scale (LSAS), which could have provided complementary information about treatment response and symptom change. Second, the VR program included only four standardized social scenarios. While this ensured consistency, it may not have captured the full range of idiosyncratic triggers for social anxiety [37]. This might raise concerns about the ecological validity of the VR environments, where the VR scenarios may not have adequately captured participants’ most salient social fears, such as informal peer interactions. Because no qualitative data were collected, this interpretation remains speculative and should be examined in future research. A further limitation concerns sample characteristics. The relatively small sample size limited statistical power to detect subtle effects and may have inflated effect sizes. Additionally, participants with psychiatric comorbidities were excluded, despite evidence that SAD frequently co‐occurs with conditions such as depression, PTSD, OCD, eating disorders, and ADHD [38]. This enhances internal validity but further limits generalizability to clinical populations. Finally, although outcome assessors and data analysts were blinded to treatment allocation, neither therapists nor patients could be blinded due to the visible differences between interventions. This potential source of bias should be considered when interpreting results. Several strengths of this study should also be highlighted. First, the randomized controlled design with a waitlist control condition strengthens causal inferences regarding treatment effects. Second, the study targeted a clinically well‐defined adolescent SAD sample using structured diagnostic interviews, enhancing internal validity. Third, outcomes were assessed at posttreatment and follow‐up, allowing examination of short‐term maintenance of treatment gains. Finally, the manualized and well‐described CBT + VRE protocol contributes to treatment transparency and replicability.

In conclusion, this pilot trial provides preliminary evidence that both CBT + VRE and CBT + IE can reduce symptoms of SAD and FNE in adolescents, with CBT + IE showing greater efficacy that persisted at 3‐month follow‐up. Given the small sample size and exploratory nature of the study, these findings should be interpreted with caution and replicated in larger RCTs. Therefore, the results primarily indicate directions for future research. The fact that CBT + VRE produced smaller effects than CBT + IE in this study has two implications: First, large‐scale trials on the efficacy of IE are necessary to allow for more reliable conclusions on its efficacy; and second, future studies should further develop more interactive and ecologically valid VR environments to determine factors of VR setups that yield the potential to enhance treatment outcomes.

## Author Contributions

**Parisa Azimisefat**: conceptualization, methodology, data collection, project administration, formal analysis, writing – original draft, writing – review and editing. **S** **amin Ravanbod**: conceptualization, methodology, data collection, writing – original draft, writing – review and editing. **Ad de Jongh**: writing – original draft, writing – review and editing. **Maryam AdliHamzehkhanlou and Farzaneh Yazdani**: data collection, review and editing. **Fateme Jamshidi**: formal analysis, writing – review and editing. **Philipp Herzog**: methodology, formal analysis, writing – original draft, writing – review and editing. **Parisa Azimisefat and Philipp Herzog**: had access to all the data, reviewed the written manuscript, provided comments, and were responsible for the decision to submit for publication.

## Funding

No funding was received for this manuscript.

## Ethics Statement

All procedures performed in studies involving human participants were in accordance with the ethical standards of the institutional and/or national research committee and with the 1964 Helsinki Declaration and its later amendments or comparable ethical standards. The trial was approved by the Research Ethics Committees of Bushehr Province University of Medical Science (Reference: IR.BPUMS.REC.1402.124) and the Iranian Registry of Clinical Trials (IRCT20210213050343N2).

## Consent

Informed consent was obtained from all individual participants and their legal guardians included in the study. Also, participants and their legal guardians signed informed consent regarding publishing their data.

## Conflicts of Interest

The authors declare no conflicts of interest.

## Data Availability

The data supporting the findings of this study are available from the Open Science Framework (OSF) data repository. Researchers interested in accessing the data can find it at the following link https://osf.io/zb268/?view_only=8bc5a170a7c84e79a0cd193e1f66aa8f. Further inquiries can be directed to the first corresponding author.
