# Correction to “Virtual Reality Exposure‐Enhanced Cognitive Behavioral Therapy (CBT + VRE) vs. CBT With Imaginal Exposure (CBT + IE) for Adolescent Patients Treatment of Social Anxiety Disorder Symptoms: A Pilot Randomized Controlled Trial”

**DOI:** 10.1155/da/9802365

**Published:** 2026-08-18

P. Azimisefat, S. Ravanbod, A. de Jongh, et al., “Virtual Reality Exposure‐Enhanced Cognitive Behavioral Therapy (CBT + VRE) vs. CBT With Imaginal Exposure (CBT + IE) for Adolescent Patients Treatment of Social Anxiety Disorder Symptoms: A Pilot Randomized Controlled Trial,” *Depression and Anxiety* 2026 (2026): 9956436, https://doi.org/10.1155/da/9956436.

In the article titled “Virtual Reality Exposure‐Enhanced Cognitive Behavioral Therapy (CBT + VRE) vs. CBT With Imaginal Exposure (CBT + IE) for Adolescent Patients Treatment of Social Anxiety Disorder Symptoms: A Pilot Randomized Controlled Trial,” there was an error in the author list. The correct author list is as follows:

Parisa Azimisefat ^∗^^†^, Samin Ravanbod^†^, Ad de Jongh, Maryam Adli Hamzehkhanlou, Fateme Jamshidi, Yousef Dehghani, Farzaneh Yazdani, and Philipp Herzog

^†^These authors have shared their first authorship.

We apologize for these errors.

